# Triosephosphate isomerase: the perfect enzyme, but how does it work?

**DOI:** 10.1107/S205225252100573X

**Published:** 2021-06-30

**Authors:** John R. Helliwell

**Affiliations:** a Department of Chemistry, University of Manchester, Manchester, M13 9PL, United Kingdom

**Keywords:** triosephosphate isomerase, isomerization, quantum refinement, QM/MM, neutron crystallography, refinement, enzyme mechanisms, structural biology

## Abstract

Insights are offered on the study by Kelpšas *et al.* [ *IUCrJ* (2021). **8**, 633–643], who have combined neutron and X-ray crystallography then QM (quantum mechanics) calculations on triosephosphate isomerase (TIM). The authors dissect three possible enzyme mechanisms of TIM that have arisen in the decades since the first X-ray crystal structure of this enzyme was published in 1975.

Nearly fifty years ago the triosephosphate isomerase enzyme, from chicken, was first determined by X-ray crystallography (Banner *et al.*, 1975 ▸). This was achieved via a multiple isomorphous replacement (MIR) phased electron-density map interpreted via a Richards box and its associated Kendrew mechanical molecular model parts (Richards, 1968 ▸). This Richards box was housed opposite my desk during my DPhil in the Laboratory of Molecular Biophysics at the University of Oxford. So, I can truly say that I once shared an office with triosephosphate isomerase!

Triosephosphate isomerase was, and is, an enzyme viewed by biochemists as ‘the perfect enzyme’. Knowles & Albery’s (1977 ▸) early review was entitled *Perfection in enzyme catalysis: the energetics of triosphosphate isomerase*. The point being that the catalytic interconversion by triosephosphate isomerase (TIM) of di­hydroxy­acetone phosphate (DHAP) and d-glyceraldehyde-3-phosphate (d-GAP) (Fig. 1 ▸) was diffusion limited and thereby made it a very fast enzyme catalyst. The enzyme gives a huge rate improvement (by 10^9^) over the reaction rate without the enzyme and a simple organic base. Knowles & Albery (1977 ▸) also remark: ‘*the structure of the enzyme at high resolution has been solved (Banner *et al.*, 1975 ▸); this confirms the existence of the unique active-site glutamate in a pocket in the enzyme, which also contains histidine and lysine residues whose detailed function will presumably emerge when the structure of the enzyme-DHAP complex is completed (Banner *et al.*, 1975 ▸).*’. As we see below, with many more X-ray crystal structures of TIM, the new study of Kelpšas *et al.* (2021 ▸), reported in this issue of **IUCrJ** and involving neutrons as a probe, was needed.

But, first a little more history. The structure of TIM (Banner *et al.*, 1975 ▸) also became famous as its 8 alpha-helices around an 8 beta-sheet-stranded barrel three-dimensional structure for its polypeptide fold was regularly seen in other enzymes. This became known as the TIM barrel. The early crystal structure studies on TIM moved beyond the native protein structure to understand the enzyme mechanism better (Alber *et al.*, 1981 ▸), already believed to involve a single base for abstracting the proton from the substrate. The X-ray crystallography undertaken by Alber *et al.* (1981 ▸) included a variety of substrate and inhibitor studies in the crystal, as well as using a flow cell and use of modest cooling to −5°C to control the enzyme. Suffice to say, TIM is active as a dimer involving mobile loops and has four catalytic residues indicated by the position of bound DHAP: Asn11, Lys13, His95 and Glu165 all from the same subunit [see Fig. 3 of Alber *et al.* (1981 ▸)]. The Glu165 was identified as the likely base.

By 2010 there were ‘at least 111 crystal structures of triose­phosphate isomerase in the PDB’ reviewed by Wierenga *et al.* (2010 ▸), a co-author of the newly published study on the enzyme (Kelpšas *et al.*, 2021 ▸). This new study utilizes combined neutron and X-ray macromolecular crystallography to determine as complete as possible structures (*i.e.* with hydrogenation details) of two complexes of the *Leishmania mexicana* triosephosphate isomerase. These complexes comprise reaction intermediate mimics, which shed light on the proton shuttling steps of the enzyme mechanism. Triosephosphate isomerase is yet another example of the case of an enzyme mechanism where controversy develops between competing models of proton movements and neutron crystallography is invoked.

Kelpšas *et al.* (2021 ▸) combined their new neutron with X-ray structures with extensive QM (quantum mechanics) calculations further deepening the understanding of triosephosphate isomerase catalysis in several ways. They describe three possible mechanisms being dissected and are explained by the authors as follows (now using the *Leishmania mexicana* amino acid sequence numbering): ‘there are (i) the so-called classical mechanism, where His95 donates a proton to the enediolate oxygen and then abstracts a proton from the other hydroxyl group of the enediol, (ii) the criss-cross mechanism where the protonated Glu167 first reprotonates the charged enediolate oxygen, followed by another proton abstraction from the other hydroxyl group of the resulting enediol. In this criss-cross mechanism the role of His95 is solely to stabilize the negative charge through strong hydrogen bonds. (iii) Another possibility, called the shuffle mechanism, is where the classical mechanism is performed in only one step, with two protons being transferred concurrently. This would avoid the formation of an intermediate where His95 would have a negative charge.’. The authors have three conclusions: ‘(i) the general base is (shown to be) definitely Glu167, (ii) there is no indication of any low-barrier hydrogen bonds and (iii) that the three suggested mechanisms are all energetically possible’. This latter point relied on the cross validation of the experimental results and QM calculations.

The paper of Kelpšas *et al.* (2021 ▸) rather masks their heroic experimental measurement efforts which they described in their earlier article (Kelpšas *et al.*, 2019 ▸). This involved successfully dealing with a low-symmetry, monoclinic, crystal system for the neutron data collection, which of course increased the total neutron beamtime required. Then, to maximize the volume of their perdeuterated crystals, they tested three crystal growth strategies: drop feeding, macroseeding and scaling up the mother-liquor volume. For one of the two studies there was an unfortunate crack in their larger crystal and they resorted to using a smaller one. There are new approaches to be harnessed. Snell & Helliwell (2021 ▸) review the alternative approach of large crystal growth of proteins for such as neutron crystallography using microgravity, so as to avoid such calamities of a cracked crystal.

In this whole story of commitment to this enzyme, its structure and its mechanism of action, there was also the detailed X-ray crystallographic study at atomic resolution by Alahuhta & Wierenga (2010 ▸), also of the highest quality, which of course is cited by Kelpšas *et al.* (2021 ▸). This involved determining protonation states via bond distances and angles and their standard uncertainties.

Overall, Kelpšas *et al.* (2021 ▸) confirms the new era of X-ray with neutron structural studies of analysing alternative enzyme mechanism choices but now also with quantum mechanics computational methods added too. I think the authors deserve a ‘congratulations’.

## Figures and Tables

**Figure 1:**
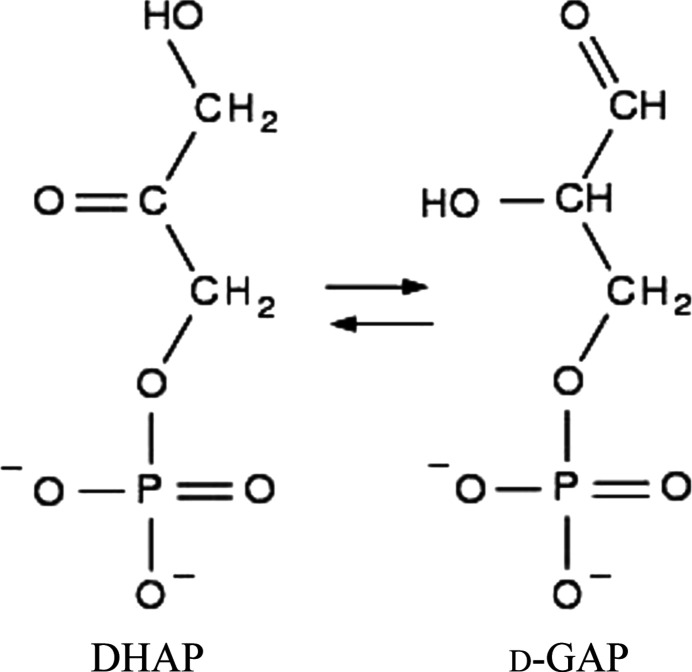
Triosephosphate isomerase catalyses the interconversion of di­hydroxy­acetone phosphate (DHAP) and d-glyceraldehyde-3-phosphate (d-GAP). Figure adapted from Alahuhta *et al.* (2008 ▸), with the permission of IUCr Journals.
